# Supplementary material for: Experiences of the COVID-19 Lockdown and Telehealth in Aotearoa New Zealand: Lessons and Insights from Mental Health Clinicians
Source: Int J Environ Res Public Health. 2023 Mar 8;20(6):4791. doi: 10.3390/ijerph20064791 (PMC10049248; doi:10.3390/ijerph20064791)
Supplement: Supplementary file 1 [file ijerph-20-04791-s001.zip › File S1.pdf]

TELEHEALTH, PROTECT THE BUBBLE: MENTAL HEALTH SERVICE DELIVERY DURING COVID-19

INTERVIEW SCHEDULE CLINICIANS

Introduction: This research study will be looking at how telehealth was helpful or not in delivering mental health outpatient services during alert levels 3 and 4 of COVID-19. When we say the word telehealth, we're thinking of video conferences/ hui (like those via Zoom) and telephone consultations. We are especially interested in topics such as how you delivered services via telehealth and how this form of service delivery influenced your relationships with clients, and other clinicians.

Everything you say today is confidential, which means I will not directly attribute anything to you, nor will your employer know that you have chosen to participate in an interview. It's your choice whether you answer questions, you can choose to stop at any time, ask for your answers to not be recorded, or for the recorder to be paused at any time. Feel free to ask questions throughout, but before we start, do you have any questions?

1. How did you find lockdown?
   Prompts: During the COVID-19 pandemic did you make any changes to how you usually manage your health or care for yourself? These could be positive or negative changes, for example, changes to diet, exercise, medicine/ alcohol taking, pharmacy services? What? Why?

2. What were your expectations around providing outpatient mental health services during COVID-19 alert levels 3 and 4?
   Prompts: What were your expectations around providing telehealth services during alert levels 3 and 4?

3. What mental health outpatient clinic do you usually work in?
4. Can you tell me about your experience with delivering services through telehealth during alert levels 3 and 4?
   Prompts: Was this your only means of service delivery? Were you more/ less busy than before?

   Did you find telehealth more effective for clients with specific conditions/ age range/ other characteristics? Which characteristics? Why?

   What form of telehealth did you use? (telephone, video conferencing e.g. Zoom)

   How did it go? How was the quality?

   How long were appointments? Was this more/ less than before?

   How was it initiated e.g. were you provided with information on how to use Zoom/ how the consultation would run? What resources did you use to learn about using [mode] of telehealth?

   How was it organised/ implemented where you work? Were there any guidelines you needed to follow?

   What worked well?

What didn't work well? (e.g. responsiveness, wrap-around services, prescription access)

What technical/ IT difficulties arose when using Zoom/ other form of telehealth and how were they overcome/ what IT support was available to you?

Before delivering services in this way over COVID-19, had you used telehealth before?

How did this influence your job satisfaction?

5. How does the service provided via telehealth differ to what you provided as part of usual care?
   Prompts: Were you able to undertake all clinical tests/ assessments/ treatment modalities? Why/ why not? How did you work around this?

   Were you able to involve whānau?

   How were able to show cultural safety/ responsiveness?
6. How did using telehealth influence your relationship with your clients? e.g. ease of access, timeliness, ability to talk with them, record conversations
7. How did providing services in this way (via telehealth) influence your ability to manage client care?
   Prompts: How did it influence collaboration between different clinicians (within your own team, within the wider service, and with other clinicians)?

   How did it influence your ability to follow up with the client?

   How did it influence the type of therapy/ service offered?

   How did it influence documentation of consultations and communication with clients?

   How did you address confidentiality while using telehealth?

   How did you maintain client privacy when using telehealth?

   How did telehealth influence your competence in caring for people with mental health conditions?

   How did it influence your confidence in caring for people with mental health conditions?
8. What elements were important to ensuring the success of telehealth consultations for you?
9. What elements do you think are important to ensuring the success of telehealth for your care team/ service provider/ client?
10. How did outcomes for clients from telehealth interactions differ from those as part of business as usual?
11. Non face-to-face consult methods such as telephone or video consults may work better for some people than others – and better for some health problems than others. In what circumstances do you think non face-to-face consults would work well for clients/ clinicians, and when do you think they wouldn't work so well?
    Prompts: When could kanohi ki te kanohi (face to face) work better?

12. Was the service 'ready' to use telehealth, why/ why not?
    <u>Prompts:</u> What were the barriers/ facilitators to use during alert levels 3 and 4 of COVID-19? e.g. client/ clinician access to the internet/ telephones.

    Was there a need for a champion/ leadership?

    Capacity within the team to use/ learn about how to use telehealth

    What are the barriers to future implementation?

    What are the facilitators to future implementation?

    What elements of telehealth service delivery would you like to see kept as part of the usual services provided? Why/ why not?

13. What suggestions would you have to improve the use of telehealth in your own practice and your teams' practice?
14. Many thanks for your time today, before we finish, is there anything else you would like to add?
15. Ask to fill in demographic form if not already completed.
